# Sanger sequencing and deconvolution of polyclonal infections: a quantitative approach to monitor drug-resistant *Plasmodium falciparum*

**DOI:** 10.1016/j.ebiom.2024.105115

**Published:** 2024-04-17

**Authors:** Hamma Maiga, Robert D. Morrison, Patrick E. Duffy

**Affiliations:** a Laboratory of Malaria Immunology and Vaccinology (LMIV), National Institute of Allergy and Infectious Diseases (NIAID), National Institutes of Health (NIH), 29 Lincoln Drive, Bethesda, MD, 20892, USA; b Institut National de Santé Publique (INSP), Ministère de la Santé et du Développement Social (MSDS), Bamako, BP: 1771, Mali

**Keywords:** Malaria, Drug resistance, Molecular marker quantification, Sequence deconvolution

## Abstract

**Background:**

Anti-malarial drug resistance in *Plasmodium falciparum* is a major public health problem in malaria-endemic regions. Although various technical improvements in sequencing methods have been introduced to identify SNPs, the conventional approach with current tools does not discriminate mixed infections, and thus can be improved for more sensitive surveillance of anti-malarial resistance to better inform control strategies.

**Methods:**

We developed a computational approach for deconvolution of chromatograms generated by standard Sanger sequencing of PCR amplicons in order to quantify molecular marker variants of anti-malarial drug resistance genes [*Plasmodium falciparum* dihydropteorate synthase (*Pfdhps*) and *P. falciparum* dihydrofolate reductase (*Pfdhfr*)]. We validated this computational approach using mixtures of V1/S and FCR3 at varying proportions between 0 and 100%, then applied it to field samples collected in Doneguebougou, Mali in 2018. We determined the mean fraction of resistance alleles in individual samples, as well as the prevalence of infections carrying resistant parasites.

**Findings:**

We observed a highly significant correlation between the predicted and measured proportions of V1/S and FCR3 alleles in mixed laboratory samples (all p < 0.001). Among field samples, the mean fraction of resistant *Pfdhps* alleles was 4.7% 431V, 95.9% 436F/A, 49.9% 437G, 0.0% 540E, 1.2% 581G and 1.5% 613S/T; corresponding prevalences were 50.0%, 100%, 72.5%, 0.0%, 25.0%, and 12.5%, respectively. The mean fraction of resistant *Pfdhfr* alleles was 0.6% 16V, 11.1% 50R, 89.0% 51I, 98.3% 59R, 74.7% 108T/N, 8.6% 140L and 8.7% 164L; corresponding prevalences were 12.5%, 75.0%, 100%, 100%, 100%, 50.0%, and 28.6%, respectively. We identified two new point mutations on the *Pfdhps* gene at codons D484T and D545N.

**Interpretation:**

Computational deconvolution of sequencing chromatograms can discriminate varying proportions of antimalarial drug-sensitive versus -resistant alleles. This cost-effective and quantitative variant-sequencing approach will be useful for population-based surveys that characterize mixed infections at the individual level to survey known and unknown mutations in *P. falciparum* drug-resistance genes.

**Funding:**

This work was supported by the Division of Intramural Research of the National Institute of Allergy and Infectious Diseases, 10.13039/100000002National Institutes of Health (NIH). HM was supported by the African Postdoctoral Training Initiative (APTI) Fellowship program jointly managed by the US NIH, The 10.13039/501100011858African Academy of Sciences (AAS) and 10.13039/100000865Bill & Melinda Gates Foundation (BMGF); Grant Reference Number: APTI-18-01.

**Research in context**

**Evidence before this study**

Molecular markers are used in epidemiological surveillance to monitor the emergence and spread of drug-resistant pathogens. These markers are comprised of either single nucleotide polymorphisms (SNPs) or concatenated SNPs in microbial genes that interact with drugs. Polyclonal malaria infections often present a mixed genotype at the loci of interest and are highly prevalent in malaria-endemic areas, particularly in Africa and Southeast Asia. In high transmission settings where polyclonal infections have been widely reported, mixed genotypes have often been binarily categorized as resistant, since any level of drug resistance might impact treatment response. To improve surveillance of antimalarial resistance control strategies, we sought an affordable solution that can quantify the proportions of all observed genotypes independently at each mutation site. Deconvolution of conventional Sanger sequencing chromatograms could provide the necessary quantification.

We did multiple PubMed and Google literature searches, starting in 2019, using search terms including: deconvolution, Sanger, chromatogram. Search results revealed that some attempts at interpreting mixtures in Sanger chromatograms have been developed previously, but none properly address the needs for surveillance of drug-resistant genotypes. Some solutions, such as TRACY, deconvolute complete chromatograms into exactly 2 alleles. Other software, such as BCV, deconvolute complete chromatograms into user pre-specified indel variants. We found no reports of software solutions that focus on independently quantifying mixed genotype calls at multiple single amino acid codon sites within Sanger sequences in a simple standalone manner.

**Added value of this study**

Our study describes a robust deconvolution method for Sanger sequencing data that is a reliable, relatively less expensive, and higher throughput for genotyping mutations associated with drug resistance. We targeted malaria genes *Plasmodium falciparum* dihydropteorate synthase (*Pfdhps*) and *P.* *falciparum* dihydrofolate reductase (*Pfdhfr*) genes in this proof-of-concept study, but the deconvolution computational approach is suitable for any protein from any species, being both gene and organism agnostic. This deconvolution method is unique in that the atomic units being modeled are the 3-base codons that code for amino acids, rather than either the entire chromatogram or just single nucleotides. The result of this deconvolution method is the relative percentages of observed protein amino acids, exactly the information needed for detailed epidemiological surveillance. Our results demonstrate quantitative discrimination of varying proportions of sensitive versus resistant alleles within samples. Compared to the binary call approach, the mean fraction or proportion determined by the deconvolution method may yield a more accurate estimate of drug-resistant parasites as a proportion of the total parasite reservoir in the community, as well as the heterogeneity of these proportions between individuals and across key groups such as children and pregnant women.

**Implications of all the available evidence**

Our computational approach to deconvolution of Sanger chromatograms at single amino acid resolution expands the set of tools for quantification of mixed mutations in polyclonal infections. This capability will improve population-based surveys to better characterize mixed infections as mean fraction or proportion at the individual level and may provide an earlier signal to public health officials of emerging resistance to better prepare for treatment policy changes.

## Introduction

In 2020, an estimated 241 million cases of malaria resulting in 627,000 deaths were reported worldwide, with over 90% of malaria cases and deaths occurring in Africa.^1^ Artemisinin-based combination therapies, the recommended first-line treatment in all countries in which malaria is endemic, have contributed considerably toward reducing malaria burden.^2^ However, the emergence of artemisinin resistance poses a serious threat to malaria control worldwide.

*Plasmodium falciparum*, the most deadly species of malaria, displays a wide variety of resistance mechanisms in the field. Current malaria control methods rely on prevention, by minimizing exposure to mosquitoes or presumptive administration of antimalarial drugs to vulnerable groups. In endemic areas where large numbers of malaria cases are presently inevitable, anti-malarial drugs are relied upon to treat the disease. *P. falciparum* resistance has been reported against every anti-malarial drug currently in use in many parts of the world.^3^

Molecular markers are used in epidemiological surveillance to monitor the emergence and spread of drug-resistant pathogens. These markers are comprised of either single nucleotide polymorphisms (SNPs) or concatenated SNPs in microbial genes that interact with drugs. Since its introduction in the 1970s, nucleic acid sequencing has allowed delineation of DNA sequences with extraordinary accuracy, and has undergone many important modifications and improvements.^4^ Improvements include longer reads (up to 1000 bp per analysis), better sequence accuracy, improved sensitivity with thermocycling protocols, full automation, higher speed, and substitution of radioactivity with fluorescent and other probes. Although various technical improvements in current sequencing methods have been introduced, including ultra-thin gel technology^5^ and capillary electrophoresis,^6^ throughput and cost of these technologies limit their use for large-scale surveillance.

*P*. *falciparum* resistance to different drugs has been associated with several alleles of *Pf* genes. *P. falciparum multi drug resistance 1 (Pfmdr1)* 86Y-184Y and *P. falciparum* chloroquine resistance transporter gene (*Pfcrt*_72C-73V-74I-75E-76T haplotype) are known as the strongest amodiaquine and chloroquine resistance markers.^7^^,^^8^ Mutations in *Pfmdr1* have been associated with reduced susceptibility to mefloquine and lumefantrine^9^ particularly *Pfmdr1* Y184F mutation associated with lumefantrine resistance.^10^ In a genome-wide association study, the E415G mutation in the *P.* *falciparum* exonuclease gene (*Pfexo_E415G)* was associated with *ex-vivo* piperaquine IC50 of parasite isolates from Cambodia. Molecular markers such as mutations in *P.* *falciparum* dihydrofolate reductase (*Pfdhfr)* and *P.* *falciparum* dihydropteroate synthetase (*Pfdhps)* are used as surveillance tools for resistance to sulfadoxine-pyrimethamine (SP).^11^^,^^12^ Resistance to pyrimethamine has been associated with numerous mutation combinations in the *Pfdhfr* gene, such as the *Pfdhfr* 51I-59R-108N/T-164L genotypes^13^ while substitutions at codons 431, 436, 437, 540, 581 and 613 of the *Pfdhps* gene may confer resistance to sulfadoxine.^14^ Sulfadoxine-pyrimethamine (SP) remains in use for intermittent preventive treatment in pregnant women (IPTp)^15^ or infants (IPTi),^16^ seasonal malaria chemoprevention (SMC),^17^ and acute malaria treatment.^18^ Monitoring of *Pfdhfr* and *Pfdhps* mutations is less complicated in geographic areas where genetic complexity of *P. falciparum* is relatively low. In most of sub-Saharan Africa (SSA), however, not only do DHFR and DHPS mutations occur in many different combinations, but most infections are “polyclonal”,^19^ meaning multiple clonal populations of parasites are present in a single blood isolate. Patients are often infected with more than one distinct parasite strain (referred to as mixed infection, multiple infection, or complexity of infection), due to bites from multiple mosquitoes, mosquitoes carrying multiple genetic types, or a combination of both.^20^ Mixed infections can lead to competition among co-existing strains and may influence disease development,^21^ transmission rates^22^ and the spread of drug resistance.^23^

Polyclonal infections are often mixed at the loci of interest (e.g., individual infections carry some parasites with mutations at specific DHFR or DHPS codons, and some parasites without mutations at those codons). Mixed infections of different genotypes are highly prevalent in malaria-endemic areas, particularly in Africa and Southeast Asia.^24^, ^25^, ^26^ In high transmission settings where polyclonal infections have been reported in greater than 50% of sampled isolates,^27^ isolates with mixed genotypes have often been classified as mutant-resistant, since any level of mutant resistance might impact treatment response.^28^, ^29^, ^30^ In some studies, polyclonal clinical samples were not included for calculation of allele prevalence and reconstruction of the various haplotypes.^31^

Many attempts at interpreting mixtures in Sanger chromatograms have been developed previously, but none properly address the needs for surveillance of drug-resistant genotypes. TRACY^32^ is a C++ command line application with a user-friendly suite of web-based front end apps that facilitate basecalling, alignment, trace decomposition, variant calling, and trace assembly. It aligns a single chromatogram of interest to a genome or FASTA sequence to identify SNPs and indels, and decomposes the given chromatogram into exactly 2 allelic sequences with an estimated allelic fraction for each overall sequence. TRACY was not designed to independently deconvolute polyclonal infections at single amino acid codon resolution where each parasite may have distinct blends of drug resistance mutations. SnpEff^33^ is a program for annotating and predicting the effect of SNPs on a genome. It requires a reference genome, and operates on variant call data more typical of Next Generation Sequencing, to predict amino acid mutations. It has no support for Sanger chromatogram data, and makes no attempt at deconvolution of mixed variants. Base-Calling with Vocabulary (BCV)^34^ is a software method that computationally deciphers Sanger chromatograms obtained from mixed DNA samples. Its stated main goal is to resolve mixed traces due to indels, and requires as input a pre-defined dictionary of possible sequences containing the indels expected to be observed. Deconvolution in BCV is to identify entire sequences, and makes no effort to quantify the relative proportions observed at individual amino acid codons. Other analytical tools, such as DECODR,^35^ TIDE,^36^ ICE^37^ and CRISP-ID^38^ are aimed at identifying CRISPR-induced indels from Sanger sequence data, but again not designed for deconvoluting single amino acid codons. MultiEditR^39^ quantifies RNA-editing events in Sanger sequences, again focused on detecting indels instead of quatifying amino acid codon mutations. TraceTrack^40^ provides for batch processing of multiple Sanger sequences to generate mutation calls against a reference sequence, but does not support deconvolution to quantify multiple codon calls within a single sequence. To date, no software solution exists that focuses on independently quantifying mixed genotype calls at multiple single amino acid codon sites within a Sanger sequence in a standalone manner.

To improve surveillance of antimalarial resistance control strategies, we implemented an approach that quantifies known and previously unknown markers of resistance in individual samples, based on the conventional Sanger sequencing chromatogram and advanced informatics tools. This approach allows the proportion of different genotypes to be estimated independently at each mutation site. We targeted *Pfdhfr* and *Pfdhps* genes in this proof-of-concept study, but the deconvolution computational approach is suitable for any protein from any species, being both gene and organism agnostic.

## Methods

### Parasite samples

*P*. *falciparum* strain V1/S is an in vitro, culture-adapted clone of the V1 strain originating in Vietnam, which shows resistance to pyrimethamine and sulfadoxine. *P. falciparum* strain FCR-3/FMG (Gambia), also referred to as FCR-3/Gambia, was originally isolated in 1976 from the blood of a human patient collected in The Gambia, West Africa. FCR-3/FMG (Gambia) was identified as resistant to chloroquine *in vitro* and *in vivo* after nearly four years of continuous culture without chloroquine pressure, and is also reported as cycloguanil-resistant and pyrimethamine-susceptible.^41^, ^42^, ^43^, ^44^, ^45^, ^46^ *Pfdhfr* and *Pfdhps* mutations for these parasite strains are summarized in Table 1. These two different parasites V1/S and FCR3/FMG were mixed in different ratios to obtain samples ranging from 0%/100%–100%/0%, respectively, in 11 mixtures with 10% successive increases/decreases in proportions (Table 2). One hundred percent corresponds to the individual strain that was not mixed. These mixture samples were generated and used for validation of the deconvolution methods.

**Table 1:** Summary of the amino acid polymorphisms of *dhfr* and *dhps P*. *falciparum* V1/S and FCR3 strains.

	*Pfdhfr*			*Pfdhps*		
	51	59	164	436	437	613
V1/S (resistant)	I	R	L	F	G	T
FCR3 (sensitive)	N	C	I	S	A	A

I, Isoleucine, R, Arginine, L, Leucine, N, Asparagine, C, Cysteine, S, Serine, A, Alanine, G, glycine, T, Threonine, F, Phenylalanine.

**Table 2:** Fractions of *P*. *falciparum* V1/S and FCR3 in strain mixtures (M1-M9).

	V1/S	M1	M2	M3	M4	M5	M6	M7	M8	M9	FCR3
Parasite mixtures (fractions)	1	0.9	0.8	0.7	0.6	0.5	0.4	0.3	0.2	0.1	0
	0	0.1	0.2	0.3	0.4	0.5	0.6	0.7	0.8	0.9	1

M, mixture of V1/S and FCR3 at different level test.

Field samples for molecular analysis were collected from adults at Doneguebougou site, Mali in 2018 during the observational study “Community Dynamics of Malaria Transmission and Mosquito Feeding in Bancoumana and Doneguebougou, Mali” (clinicaltrials.gov ID NCT03304704). One mL of venous blood was collected in EDTA anticoagulant tubes among participants, and parasitized blood samples were stored at −80 °C until analysis.

### DNA extraction

QIAamp DNA blood mini kits (Qiagen, Valencia, CA USA) were used according to the manufacturer's instructions to extract genomic DNA from 200 μL EDTA whole blood which was then eluted in 60 μL of buffer AE (10 mM Tris-Cl and 0.5 mM EDTA). The concentration and purity of DNA were quantified using a NanoDrop2000 UV (Thermo Fischer, Wilmington, DE, USA).

### PCR and sequencing

*Pfdhfr* and *Pfdhps* fragments were amplified by PCR. Briefly, Dhfr_Fwd (5′TTTATATTTTCTCCTTTTTA3′) and Dhfr_Rev (5′CATTTTATTATTCGTTTTCT3′) oligonucleotides were used for single amplification of *Pfdhfr* sequences, producing a 681 bp fragment containing key mutations N51I, C59R and I164L. For *Pfdhps* sequences, dhps_Fwd (5′GGAATATTAAATGTTAATTATGATTCT3′) and dhps_Rev (5′CGTCATGAACTCTTAT-TAGATCTACCT3′) were used for single amplification, producing a 986 bp fragment containing mutations S436 F/A, A437G and A613 S/T, previously associated with parasite responses to sulfonamides (e.g., sulfadoxine, sulfamethoxazole, and sulfathiazole).

The reaction comprised 5 μl of DNA, 0.5 μl of each primer (dhfr_Forward or dhps_Fwd and dhfr_Reverse or dhps_Rev with final concentration 0.5 μmol/L each), 25 μl of Q5 High/Hot-Fidelity 2X Master Mix and 29 μl of Nuclease-free Water. The reaction was cycled as follows: 5 min at 95 °C and then 44 repeated cycles of 30 s at 92 °C, 30 s at 45 °C and 45 s at 65 °C followed by 15 min at 72 °C. PCR products were purified by QIAamp DNA blood mini kits and concentration was measured using a NanoDrop2000 UV. The PCR products were sequenced by Eurofins Genomics Company.

### Drug resistance calls

Mutation results can be reported by either of two different methods that impact the interpretation of drug resistance studies. The quantitative method (mean fraction of resistant allele in individual samples) gives the numeric proportion for the mutation of interest, while the conventional method (prevalence of resistant parasites in a population of samples) is instead based on binary calls, where any amount of detection of resistant allele labels the entire sample resistant, while a sample is only called susceptible if it has a total absence of any amount of resistant allele. Because the conventional method omits the fractional specificity of mixed infections, these two reporting methods may produce divergent findings.

### Chromatogram deconvolution

**Fig. 1:**
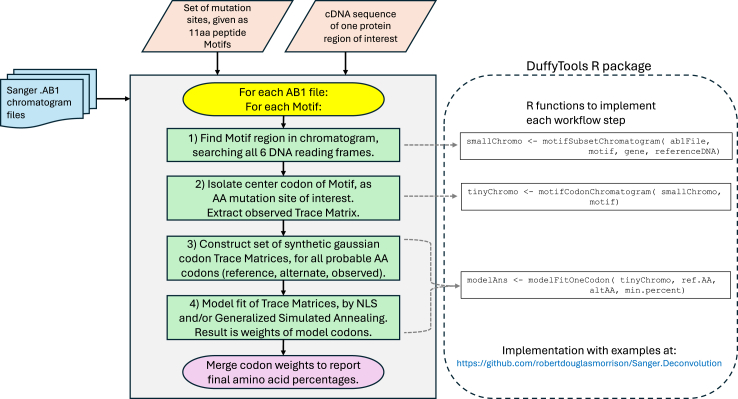
**Flowchart of deconvolution approach**. The workflow of the deconvolution method is shown both descriptively, showing data inputs and processing steps to produce the mutation proportion calls, and as the actual R function calls to implement the workflow on an example chromatogram.

Quantification by deconvolution of resistance mutation sites within a chromatogram used a multistep workflow, implemented as part of the DuffyTools R package (https://github.com/robertdouglasmorrison/DuffyTools), and visualized as a flowchart in Fig. 1. First, the raw binary ABI chromatogram file was imported into R using the sangerseqR package from Bioconductor,^47^^,^^48^ to extract the numeric trace matrix of signal intensities for all 4 base channels along the entire extent of the chromatogram, as well as the list of nucleotide base calls and their peak center locations within the trace matrix. The base call sequence was saved in both its original and reverse complement forms and translated into all 6 reading frames of possible protein sequence. The collection of DNA and amino acid calls, spectral peak center locations, and the full trace matrix of intensities in all 4 channels constituted the starting dataset for all subsequent mutation quantification steps. To facilitate automated assessment of the chromatogram sequence, each base call was augmented with a confidence score metric, similar to the Phred quality Q scores^49^ that uses peak height, shape and uniqueness across the 4 channels to differentiate clean base calls from chromatogram noise. All the above steps are encapsulated into the DuffyTools function *loadChromatogram()* (Figure S1).

The next step was to locate and isolate the small region of the chromatogram that contains the mutation site of interest, in a manner that tolerates the presence of unknown mutations that might be present in field parasites. Evaluations using mixtures of lab line parasites suggested that an 11 amino acid fragment motif, centered on the mutation site of interest, from the 3D7 reference protein, gave the best performance for finding the correct region of interest across a wide variety of chromatograms, proteins, and mutation sites. For example, to find the A581G mutation site in a chromatogram from *Pfdhps*, the motif ‘IGLGFAKKHDQ’ provided 5aa of hopefully conserved flanking context on both sides of the mutation of interest. Given this protein fragment motif, the raw chromatogram was searched in all 6 reading frames using the *pairwiseAlignment()* function from the Biostrings^50^ package, to find the best scoring sequence match location while tolerating amino acid mismatches. Using that location and reading frame information, a small chromatogram subset was extracted from the original raw data containing only the 11 amino acid region that was found, in the correct strand and reading frame, discarding all extraneous portions of the trace matrix, peak locations, and base calls; positioning the 3-base codon of interest for the wanted mutation site at the exact center of this new smaller chromatogram. All the above methodology is encapsulated into DuffyTools function *motifSubsetChromatogram()* (Figure S2).

**Fig. 2:**
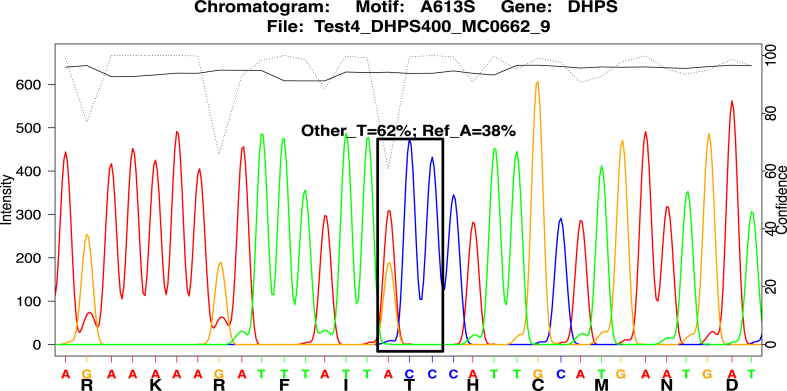
**Deconvolution model fitting result of *Pfdhps* A613 S/T**. The final deconvolution model fit amino acid call for *Pfdhps* site A613S, visualized on the motif subset chromatogram. In this example from M4 mixture of V1S and FCR3 (Table 2), the method estimates 62% of DNA encodes Thr (ACC) and 38% encodes Ala (GCC), consistent with expected 60/40%.

The final deconvolution quantification step was to model and measure the mutation site. First, the raw trace matrix of the observed 3bp DNA codon at the mutation site was extracted from the center of the motif chromatogram, using function *motifCodonChromatogram().* Next, the collection of candidates 3bp codons for inclusion in the model was generated by gathering all possible codons that code for up to 3 different amino acids: the reference amino acid, the expected alternative mutation amino acid, and the observed chromatogram-called amino acid at the codon site. Each of these candidate codons was modeled as an idealized trace matrix containing 3 gaussian shaped synthetic nucleotide peaks, using peak width and separation parameters measured from the observed chromatogram. This effectively makes a collection of minute synthetic trace matrices representing the signal intensity profile each candidate codon would generate (Figure S3). For example, the candidate ATG codon for methionine is constructed as a synthetic trace matrix with one fixed width gaussian peak in the 1st third of the A channel, a fixed width gaussian peak in the 2nd third of the T channel, a fixed width gaussian peak in the 3rd third of the G channel, and no peaks anywhere in the C channel. Lastly, these candidate codons and the observed chromatogram codon were passed to a model fitting step, using both a Nonlinear Least Squares (NLS)^51^ optimization method and a simulated annealing optimization method using the GenSA package.^52^ NLS was the preferred model fit algorithm, but because the observed data are sparse (only 3 DNA bases of spectral peaks), may contain noisy malformed peak artifacts, and the number of candidate codons might be large, the NLS method might fail to successfully converge. If NLS failed, the GenSA method was used instead, being more robust but at the expense of longer computational time and potentially returning a less optimal fit. The goal of the model fit is to explain the observed 3-nucleotide trace matrix as a linear additive blend of all the synthetic candidate codon trace matrices, adjusting the non-negative amplitude of each candidate codon as needed to minimize the residual error between the one observed and the sum of all synthetic trace matrices. The result of the model fit step is the subset of candidate codons having non-zero signal amplitudes (weights), as the best explanation of the observed codon chromatogram (Figure S4). All candidate codons with zero weights are discarded, and only those codons having non-zero weights were translated back to amino acids, scaling their weights proportionally to sum to 100%, giving the final proportion of amino acid calls that best model the observed raw data (Fig. 2). The methodology intentionally includes all possible codons in the model, and lets the model fit determine which small subset of codons best explain the observed data. Any attempt to down-select potential codons for inclusion prior to the model fit step would be based on either user choice or inspecting the observed data, both of which would introduce potential bias into the model. All the above deconvolution methodology and model fitting is encapsulated into the DuffyTools function *modelFitOneCodon()*.

### Algorithm availabilty

The entire multistep algorithmic approach has been packaged into publicly available R markdown scripts suitable for RStudio^53^ that can process a single chromatogram or batch process large sets of chromatograms. See https://github.com/robertdouglasmorrison/Sanger.Deconvolution The GitHub page contains all scripts, instructions, a clear description of the algorithm, example input chromatograms, and example result files in both HTML and CSV formats. Clone or download using GitHub's Code menu button.

### Ethics

The study protocol was approved by the ethical committees of the Faculty of Medicine and Odonto-stomatology (FMOS)/Faculty of Pharmacy (FAPH)/University of Sciences, Techniques and Technologies of Bamako (USTTB) of Mali and US-NIH (clinicaltrials.gov ID NCT03304704). Community permission was obtained before to the study. Individual, written, informed consent was obtained of each volunteer prior to screening.

### Statistical analysis

Field samples were selected randomly with blinding from parasite-positive visits. Data were collected on sequencing printout and entered into a Microsoft Excel™ database 2010 (Microsoft Inc., Washington, WA). Statistical analysis was performed using RStudio (R version 3.3.0). The mix at different proportion from V1/S to FCR3 was calculated with 95% Confidence interval [CI]. Logistic regression model was used for comparisons as appropriate with statistical significance set at p-value <0.05.

### Role of funders

The funders had no role in study design, data collection, data analyses, interpretation, writing of the manuscript, or the decision to submit for publication.

## Results

### Laboratory samples

We validated the approach using mixtures of V1/S and FCR3 at varying proportions between 0 and 100% in increments/decrements of 10%. We interpreted the results as the mean fraction of resistance alleles in all samples or as the prevalence of samples carrying any detectable *Pfdhps* and *Pfdhfr* resistance genes.

#### Mean fraction and prevalence of *Pfdhps* alleles

**Fig. 3:**
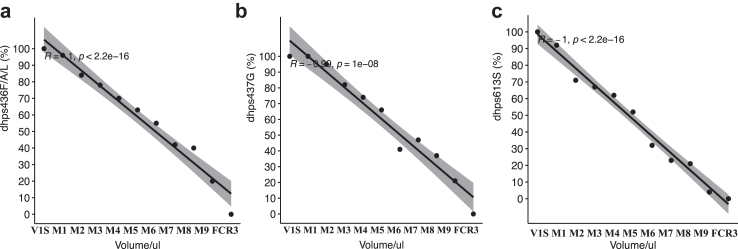
**Correlation of the quantified (%) versus predicted (Volume/ul) mutant allele of *Pfdhps* in mixture of V1S and FCR3 parasites**. Correlation of the phenotype resistance proportion with mixture volume. V1/S and FCR3 parasites represent 100% mutant and sensitive, respectively. (3a) Mutant allele of *Pfdhps* 436F/A proportion, (3b) mutant allele of *Pfdhps* 437G proportion and (3c) mutant allele of *Pfdhps* 613S proportion.

As expected, the proportion of individual resistance alleles decreased from V1/S and M1 to M9 and FCR3, for S436F (Figure S5a), A437G (Figure S5b) and A613 S/T (Figure S5c) that followed the inverse order. The mean fraction of VI/S for resistant *Pfdhps* alleles was 60.3% 436F/A, 60.3% 437G and 47.6% 613S/T, respectively (Figure S6a). When applying the binary call approach that is common in field research, the resistance mutant prevalence was estimated at 91.0% for each codon (Figure S6b). We observed a significant correlation between the measured fraction of resistant *Pfdhps* alleles 436F/A, 437G and 613S/T versus the predicted fraction based on the mixed volumes (p < 0.001, Fig. 3). The results showed 100% of the *Pfdhps* K540 sensitive and *Pfdhfr* 108T/N resistant (data not shown).

#### Mean fraction and prevalence of *Pfdhfr* alleles

**Fig. 4:**
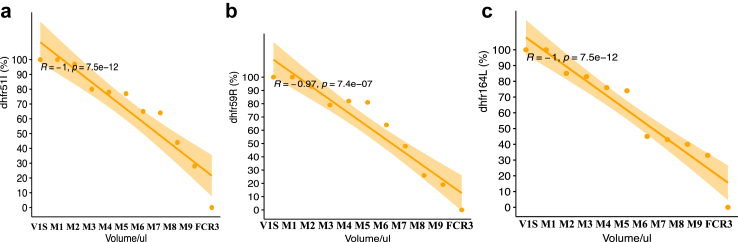
**Correlation of the quantified (%) versus predicted (Volume/ul) mutant allele of *Pfdhfr* in mixture of V1S and FCR3 parasites.** Correlation between mixture volume and phenotype resistance proportion. (4a) Mutant allele of *Pfdhfr* 51I proportion, (4b) mutant allele of *Pfdhfr* 59R proportion and (4c) mutant allele of *Pfdhfr* 164L proportion.

The proportion of individual sensitive alleles of *Pfdhfr* in a) N51I b) C59R; and c) I164L was decreased from FCR3 and M9 to M1 and VI/S that followed the inverse order (Figure S7). The mean fraction of V1/S for resistant *Pfdhfr* alleles was 66.6% 51I, 62.0% 59R and 61.7% 164L, respectively (Figure S8a). The total prevalence of V1/S presented 91.0% for each resistant *Pfdhfr* alleles (51I, 59R and 164L) by binary call approach (Figure S8b). We observed a strong linear correlation between the proportion predicted by the mixed volumes and the measured proportion of resistant *Pfdhfr* alleles (p < 0.001, Fig. 4).

#### Comparison against published deconvolution tool TRACY

To evaluate how the method differs from existing deconvolution tools, we submitted the chromatogram of one of the DHPS lab mixtures (60% V1/S, 40% FCR3, as shown in Fig. 2, and Fig. S1 through Fig. S4) to the Indigo deconvolution tool of the TRACY^32^ suite. TRACY requires a reference DNA sequence as a comparitor, so DHPS from *P.falciparum* reference isolate 3D7 was given. The result returned by TRACY declared that the chromogram was best explained as being 64% reference 3D7 and 35% an alternative sequence that contained a mashup of various mutations from both V1/S and FCR3. At A613 S/T, where the known mutation call is 60%T/40%A, TRACY called 64%A/35%T. At mutation site S436F, where the known mutation call is 60%F/40%S, TRACY called 64%S/35%F. Overall, TRACY's proportion magnitudes were close to correct, but the major/minor variant was inverted in both cases. Furthermore, the TRACY output reveals it was incorporating different signal proportions at 18 separate nucleotide locations along the high quality portion of the chromatogram, to average all those differences into exactly 2 global proportions that describe the entire chromatogram. TRACY was unable to separately evaluate each mutation site of interest, and was confounded by requiring inclusion of every other mutation and chromatogram noise detected along the region into its result, regardless of their role (or lack thereof) in drug resistance.

### Field samples

We applied the quantitative method on eight field samples to determine the mean fraction of resistance alleles among human isolates, as well as their prevalence defined as any detectable *Pfdhps* and *Pfdhfr* resistance genes.

#### Mean fraction and prevalence of *Pfdhps* alleles

**Fig. 5:**
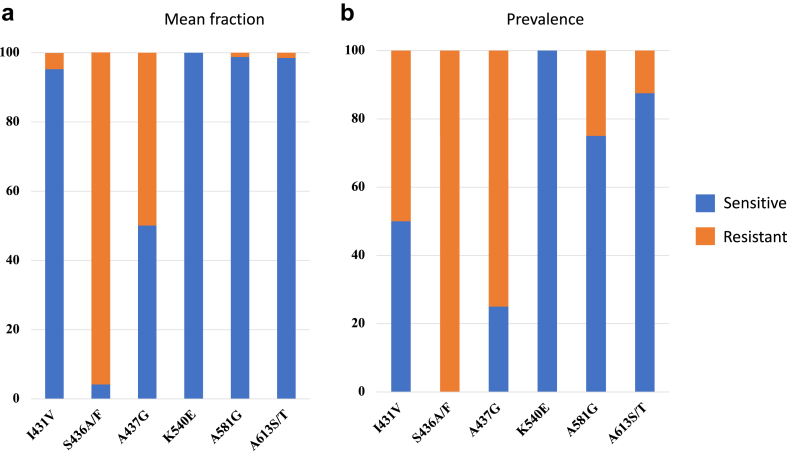
**Prevalence and Mean fraction of *Pfdhps* alleles at codons 431, 436, 437, 540, 581, and 613 of field samples**. Presents a binary call result (S = sensitive; R = resistance); mean fraction presented in panel (5a), and prevalence presented in panel (5b).

The mean fraction of resistant *Pfdhps* alleles was 4.7% 431V, 95.9% 436F/A, 49.9% 437G, 0.0% 540E, 1.2% 581G and 1.5% 613S/T, respectively (Fig. 5a). By the binary call approach, the prevalence of mutant was 50.0%, 100%, 72.5%, 0.0%, 25.0% and 12.5% for the same resistant *Pfdhps* alleles, respectively (Fig. 5b). Mixed alleles (wild-type and mutant) were identified in 50.0%, 25.0%, 37.5%, 0.0%, 25.0% and 12.5% of samples at different *Pfdhps* loci (I431V, S436 F/A, A437G, K540E, A581G, and A613 S/T), respectively (Figure S9).

#### Mean fraction and prevalence of *Pfdhfr* alleles

**Fig. 6:**
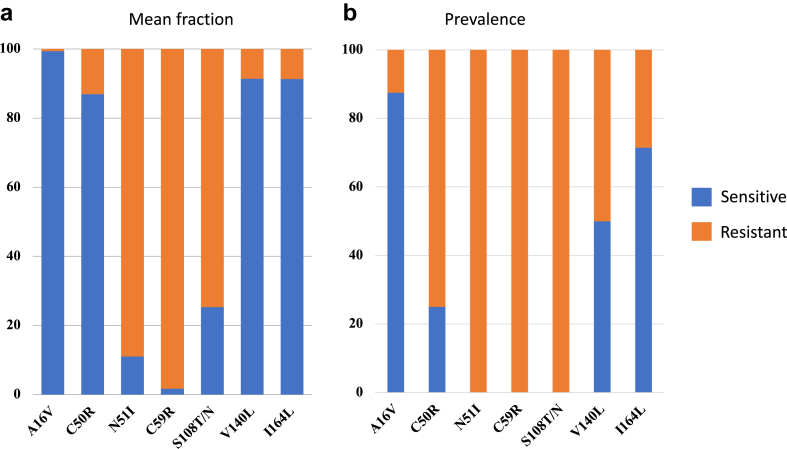
**Prevalence and mean fraction alleles of *Pfdhfr* at codons 16, 50, 51, 59, 108, 140 and 164 of field samples**. Presents a binary call result (S = sensitive; R = resistance); mean fraction presented in panel (6a), and prevalence presented in panel (6b).

The mean fraction of resistant *Pfdhfr* alleles was 0.6% 16V, 11.1% 50R, 89.0% 51I, 98.3% 59R, 74.7% 108T/N, 8.6% 140L and 8.7% 164L, respectively (Fig. 6a). The prevalence of mutant was 12.5%, 75.0%, 100%, 100%, 100%, 50.0% and 28.6% for the same resistant *Pfdhfr* alleles, respectively (Fig. 6b). Mixed alleles (wild-type and mutant) were identified in 12.5%, 75.0%, 50.0%, 28.6%, 100%, 50.0% and 28.6% of samples at different *Pfdhfr* loci (A16V, C50R, N51I, C59R, S108 T/N, V140L and I164L), respectively (Figure S10).

## Discussion

DNA-sequencing is the gold standard for assessing molecular resistance to antimalarial drugs^54^ and proportions of *P.* *falciparum* genotypes are of major importance for epidemiological surveys. However, the conventional method using binary calls for antimalarial resistance alleles within samples fails to characterize the proportion of a particular genotype in a mixed sample. Pyrosequencing is a quantitative method with a superior limit of detection (proportion of resistant alleles) compared to Sanger sequencing.^55^ However, the pyrosequencing method requires the presence of at least 30% mutant DNA in mixed samples to detect *Pfdhfr* at codon 164L and uses many primers with high cost per sample.^56^ For example, the cost estimate for pyrosequencing is $2.28 per SNP^57^ and for restriction fragment length polymorphism (RFLP) is $6.90 per SNP.^58^ Our approach enables estimation of the relative burden of individual molecular markers of SP drug resistance, and will be more cost-effective and sensitive to detect the emergence and spread of known and unknown resistance alleles which threaten public health.

In this paper, we report a bioinformatic approach to quantify the molecular markers of SP using sequence chromatograms, as a cost-effective alternative to guide decision-makers in the face of spreading resistance to anti-malarial drugs. Commonly used tools yield a binary or categorical call to define the molecular markers of antimalarial resistance, and do not allow quantitative assessment of variants within mixed infections. To improve surveillance of antimalarial resistance control strategies, our approach allows the proportion of different genotypes to be estimated from Sanger sequencing chromatograms. We evaluated the performance of the method to correctly estimate the true proportion of different alleles, first using laboratory parasite mixtures with known fractions of resistant and wild-type alleles.

Using fixed ratios of laboratory parasites, our study demonstrated the ability of our deconvolution method to detect a minor allele at a proportion as low as 10%. As expected, the standard approach that assigns a binary call would have designated all of our laboratory parasite mixtures as resistant at all *Pfdhps* and *Pfdhfr* loci,^46^^,^^47^ while the mean fraction of resistance markers using the quantitative approach was nearly 50% for all loci. We observed a highly significant correlation between the known proportion of each allele and the proportion measured by sequencing and deconvolution at all loci.

After qualifying our methods on laboratory samples, we applied them to field samples collected in Mali in 2018. In the field samples, the mean fraction of resistant *Pfdhps* alleles (431V, 436F/A, 437G, 540E, 581G, and 613S/T) and of resistant *Pfdhfr* alleles (16V, 50R, 51I, 59R, 108T/N, 140L and 164L) demonstrate quantitative discrimination of varying proportions of sensitive versus resistant alleles within samples. Polyclonal infection is a common phenomenon in malaria-endemic areas; Diakité et al. showed a very high genetic diversity in *P. falciparum* isolates in Mali consistent with previous findings throughout Africa.^59^^,^^60^ Complexity of infection has been associated with intensity of malaria transmission.^61^^,^^62^

Our deconvolution method is based on standard sequencing while being more rapid, cost-effective by sample for all mutations ($10 for six or seven SNPs we studied in the genes), and sensitive (down to at least a 10% proportion of the resistant allele in our sample mixture studies) compared to pyrosequencing and restriction fragment length polymorphism (RFLP). A pyrosequencing protocol was developed as a rapid and reliable method to identify the mutations of *Pfdhfr* (at codons 16, 51, 59, 108, 140, and 164) and *Pfdhps* (at codons 431, 436, 437, 540, 581 and 613) genes of *P. falciparum*,^55^ but used ten and twelve primers to amplify *Pfdhfr* and *Pfdhps* genes, respectively, compared to four primers used in our deconvolution method for the same genes and all mutations. RFLP is a common method used for each SNP, using 32 primers for *Pfdhfr* (at codons 16, 51, 59, 108, 140, and 164) and 24 primers for *Pfdhps* (at codons 431, 436, 437, 540, 581 and 613) gene amplification. Pyrosequencing and RFLP methods are more time-consuming compared to the deconvolution method.

Zhou et al. noted that pyrosequencing and RFLP methods showed similar levels of sensitivity in detecting the mutant forms of *Pfdhfr* SNPs, except for codon 164 in mixed samples.^55^ Also, pyrosequencing required the presence of at least 30% mutant DNA in mixed samples to detect the 164L mutation, while RFLP was sensitive enough to detect this mutation with as little as 15% mutant DNA. Repeated RFLP experiments further escalated the cost of reagents for this procedure. The deconvolution method was sensitive enough to detect all mutations at proportions as low as 10%. The reagents used in the deconvolution method were relatively inexpensive compared to those for RFLP and pyrosequencing. RFLP was at least threefold more expensive than pyrosequencing in reagent costs alone, and reagent costs for our deconvolution method are inexpensive compared to those for pyrosequencing.

Our findings are limited to analysis of *Pfdhps* and *Pfdhfr* genes, above all the K540E and S108 T/N mutations that are most important in sub-Saharan Africa. While we did not analyze *Pfcrt, Pfmdr1,* or *PfK13* genes in this study, we have developed the same method for other Pf genes (e.g., *MDR*, *CRT*, *CYTB*, *HRP2*, among others), and our method is not limited to “pairs of genes” but could be used for any number. Our pilot study was limited to a relatively small number of field samples, but also includes data from mixtures of laboratory isolates to demonstrate the linear range of quantitation. Further, resistance can be related to changes in the copy number of genes encoding the drug target or transport proteins involved in drug influx/efflux.^63^ Our approach has not been designed to assess copy number.

The deconvolution method has the benefit that it uses a single sequencing reaction and can detect any potential new mutations that have not been previously described within the PCR-amplified region. Existing published methods such as TRACY fail to deconvolute results at single mutation resolution. For example, during our study we have discovered two new point mutations on the *Pfdhps* gene at codons D484T and D545N. We are interested in future to assess any role for these identified SNPs in antimalarial resistance. This advantage of the deconvolution method equally pertains to resistance markers for other antimalarial drugs. An important future application could be to monitor artemisinin resistant parasites as they spread across Africa including emerging variants of the kelch 13 gene.^64^

Other benefits of this deconvolution method derive from its flexibility. It is organism agnostic, taking only the reference DNA sequence of the expected PCR-amplified region and a list of amino acid motifs as the only required inputs other than the chromatograms. The list of motifs is fully user specified, so any coding region of any protein can be evaluated by this method. Empirical simulations using several *P.* *falciparum* cDNA sequences suggested that 11aa was an ideal motif length, providing enough local context around the mutation site of interest even when multiple nearby residues were also mutated, such that the search in all 6 reading frames always finds at most a single high scoring match to the motif. Shorter or longer motifs can be used, with the caveat that too short a motif may identify multiple non-unique matches across the 6 reading frames, while a too long motif may extend past the limits of the chromatogram sequence or span possible indel regions of the protein and thus fail to identify a single high scoring match. The only cause for this deconvolution method to fail is when the motif cannot be found in any of the 6 reading frames. The two most likely reasons a motif fails to be found are: noisy chromatograms without clear singular peak calls in the region of interest, or that the isolate being sequenced is highly mutated in that region such that the expected motif is not present. The user is fully free to adjust the motif length or sequence for each mutation site of interest. This use of user-specified amino acid motifs allows the deconvolution method to locate the exact mutation sites of interest anywhere in the chromatogram in a fully automated method.

While sequencing capabilities in Africa have been hindered by logistical and infrastructure limitations in the past, many labs in Africa now have sequencing platforms, making this technology accessible for local scientists. Our technique offers practical advantages and is relatively easy to implement for surveillance of drug-resistant *P. falciparum* markers in the malaria elimination context. We know that molecular markers associated with antimalarial drug resistance, when validated, are invaluable to monitor geospatial distribution of resistant parasites in real time. The prevalence of molecular markers of drug resistance is often a good indicator of the level of clinical resistance.^63^

Next Generation Sequencing (NGS) is a leading choice for many types of sequence variant discovery, but less ideal for this goal of easy low-cost drug-resistance surveillance. NGS is more expensive than Sanger sequencing, and requires more highly specialized technicians, equipment, and computational infrastructure. Individual NGS reads are discrete, having only ACGT fixed calls. Any attempt to quantify proportions of alleles requires aligning large numbers of reads to a reference genome to build consensus pileups and then using variant calling software to detect the relative proportions of each base call. One distinct advantage of NGS methods is in detecting the phasing of nearby mutations. For mutations that are closer together than the length of the NGS reads, by inspecting individual reads one could count how often particular mutation pairs were seen in the same DNA fragment. In our Sanger chromatogram approach, phasing can only be roughly estimated from the amino acid proportions.

In conclusion, the present study describes a deconvolution method that is a reliable, relatively less expensive, and higher throughput alternative for genotyping mutations of *Pfdhfr* and *Pfdhps* associated with drug resistance. Our results demonstrate quantitative discrimination of varying proportions of sensitive versus resistant alleles within samples. Compared to the binary call approach, the mean fraction determined by the deconvolution method may yield a more accurate estimate of drug-resistant parasites as a proportion of the total parasite reservoir in the community, as well as the heterogeneity of these proportions between individuals and across key groups such as children and pregnant women. This relatively simple approach for population-based surveys that characterizes mixed infections at the individual level may provide an earlier signal of emerging resistance to better prepare public health officials for treatment policy changes.

## Contributors

H.M. and P.E.D. conceived the study. H.M conducted the laboratory study and wrote the first draft of manuscript, which was revised by P.E.D for initial submission. R.D.M. designed and implemented the chromatogram deconvolution algorithms, performed statistical data analysis, and edited the manuscript. All authors accessed and verified the underlying data. All authors reviewed, edited, and approved the final manuscript version after review, and agreed to the published version of the manuscript.

## Data sharing statement

All data are included in the manuscript, figures, and supplemental data, and are available at the time of publication, from the authors upon reasonable request.

## Declaration of interests

We declare no competing interests.
