# Supplementary material for: Sanger sequencing and deconvolution of polyclonal infections: a quantitative approach to monitor drug-resistant Plasmodium falciparum
Source: eBioMedicine. 2024 Apr 17;103:105115. doi: 10.1016/j.ebiom.2024.105115 (PMC11031737; doi:10.1016/j.ebiom.2024.105115)

Supplementary Figure 1. Initial interpretation of full length chromatogram.

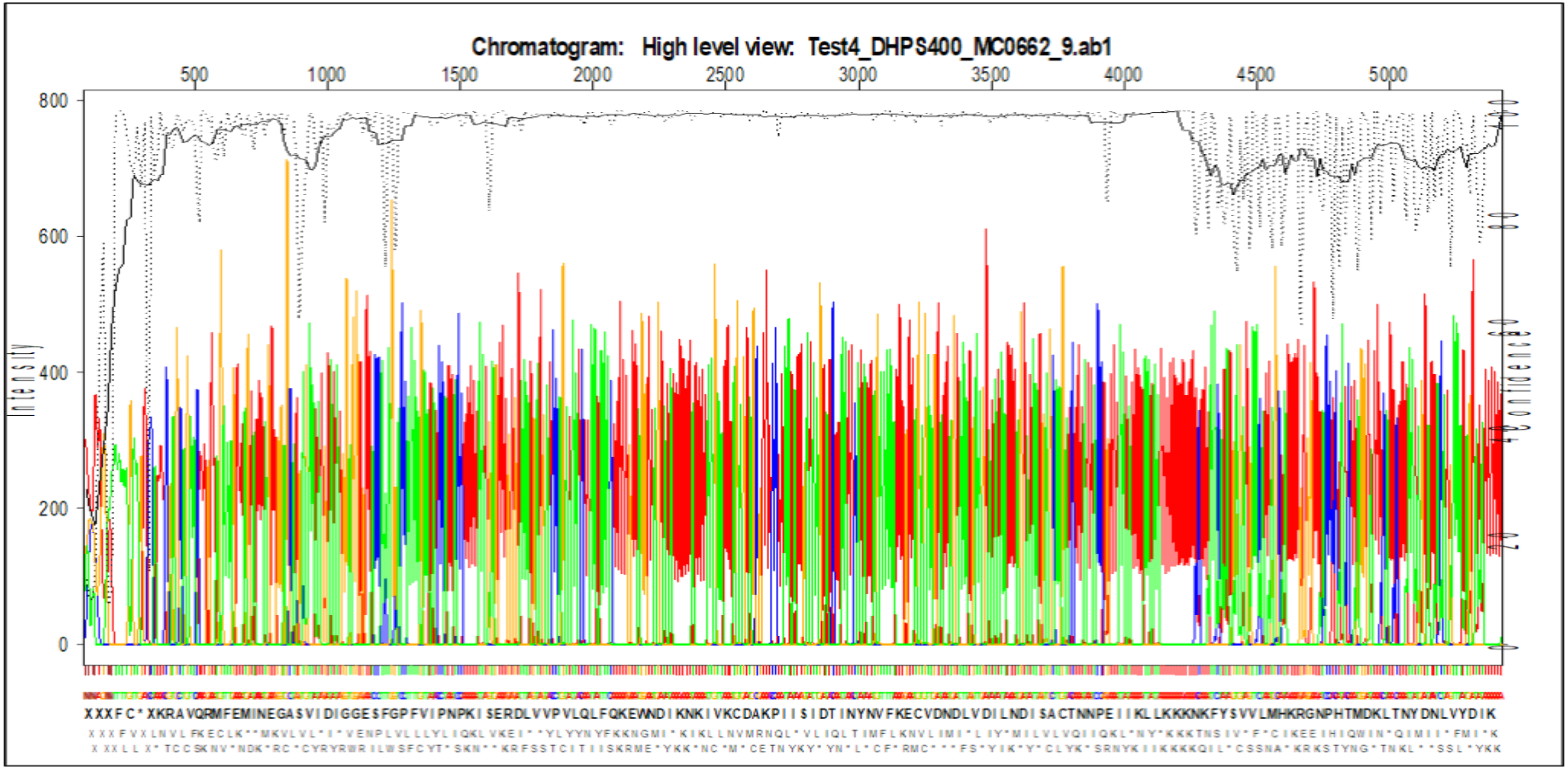

Supplementary Figure 2. Chromatogram motif subset centered on mutation site of interest.

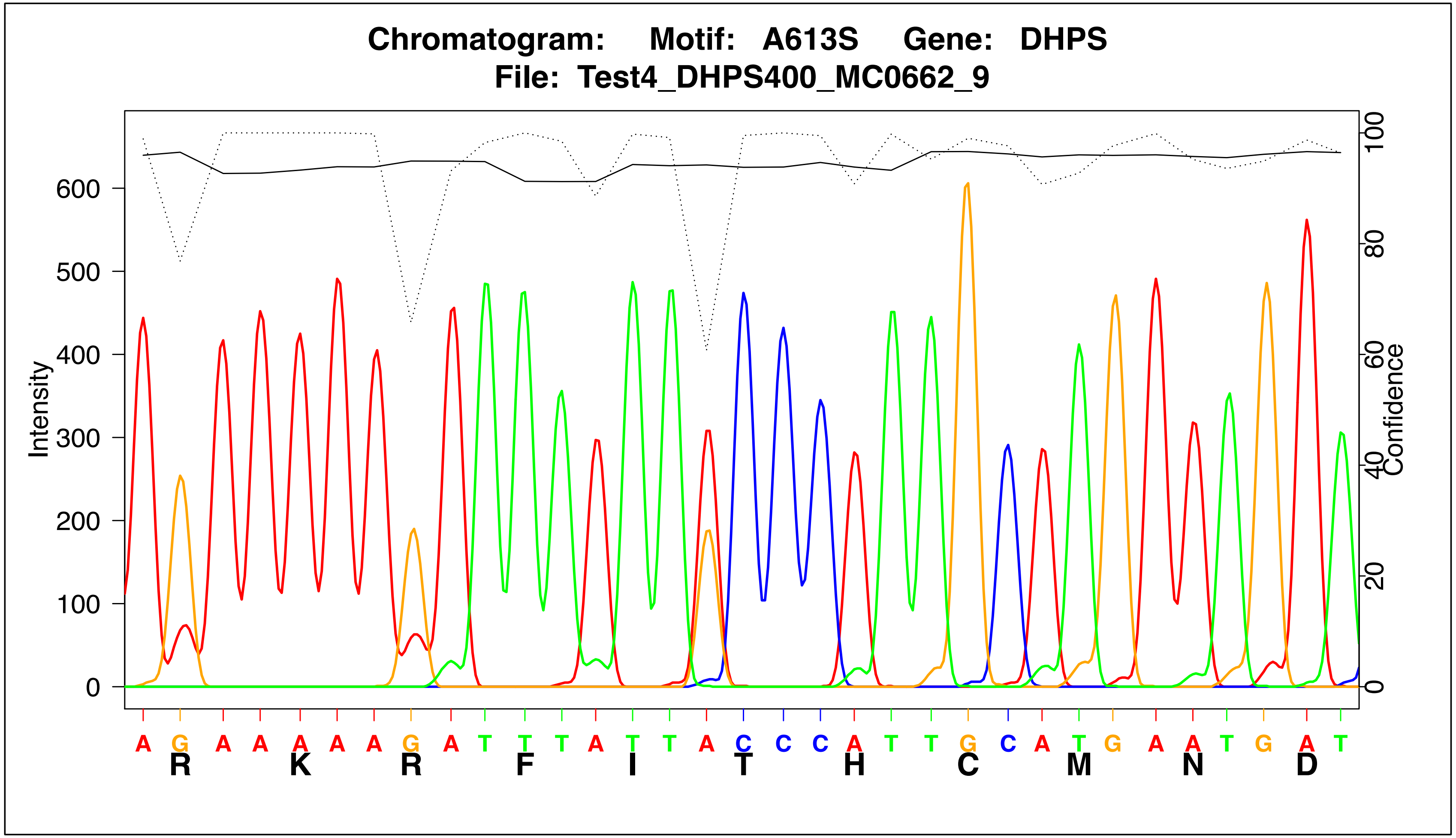

Supplementary Figure 3. Deconvolution model inputs: observed and all possible candidate codons.

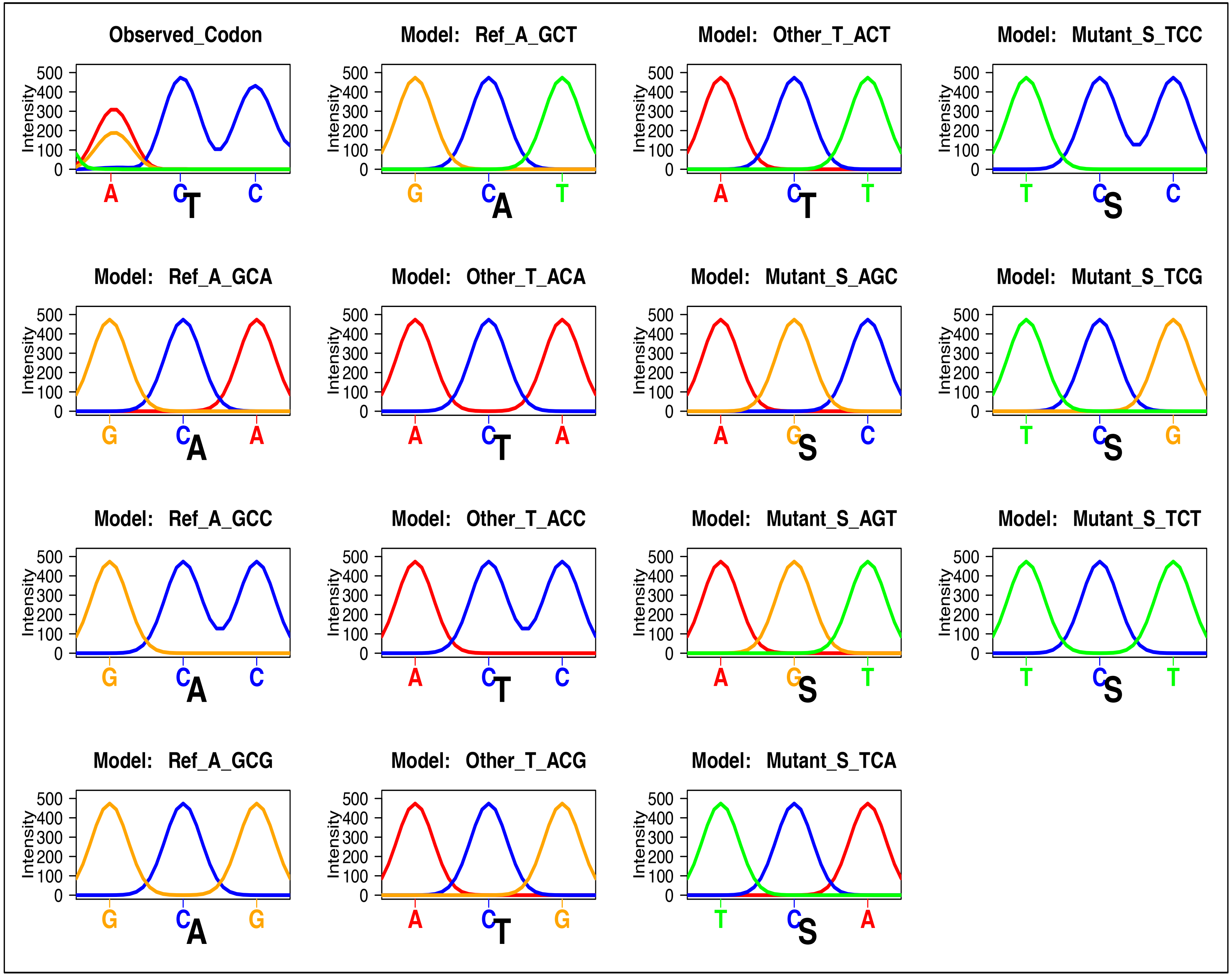

Supplementary Figure 4. Deconvolution model output: best fit codons and signal amplitudes.

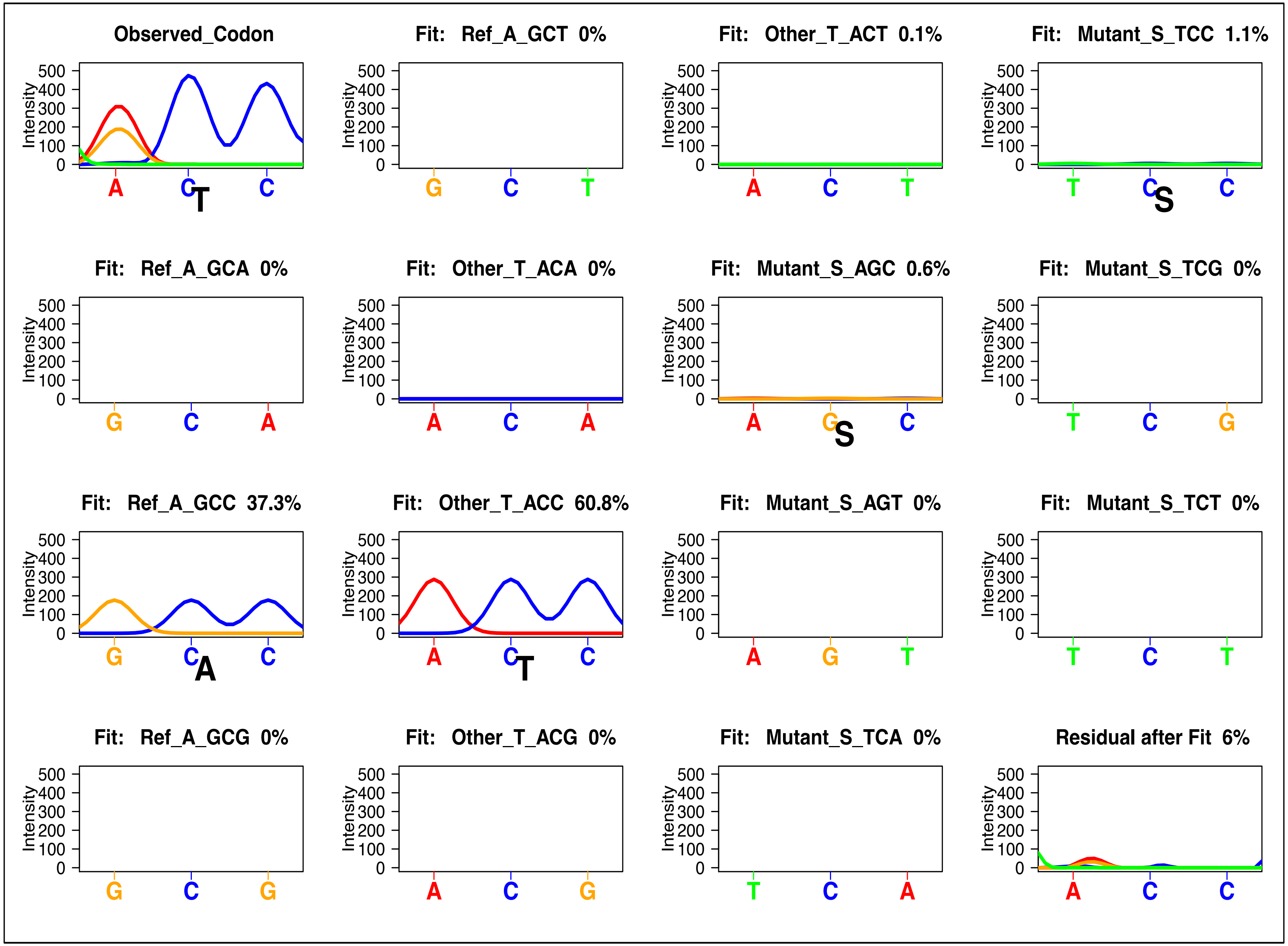

Supplementary Figure 5. Proportion of alleles of Pfdhps at codons 436, 437 and 613

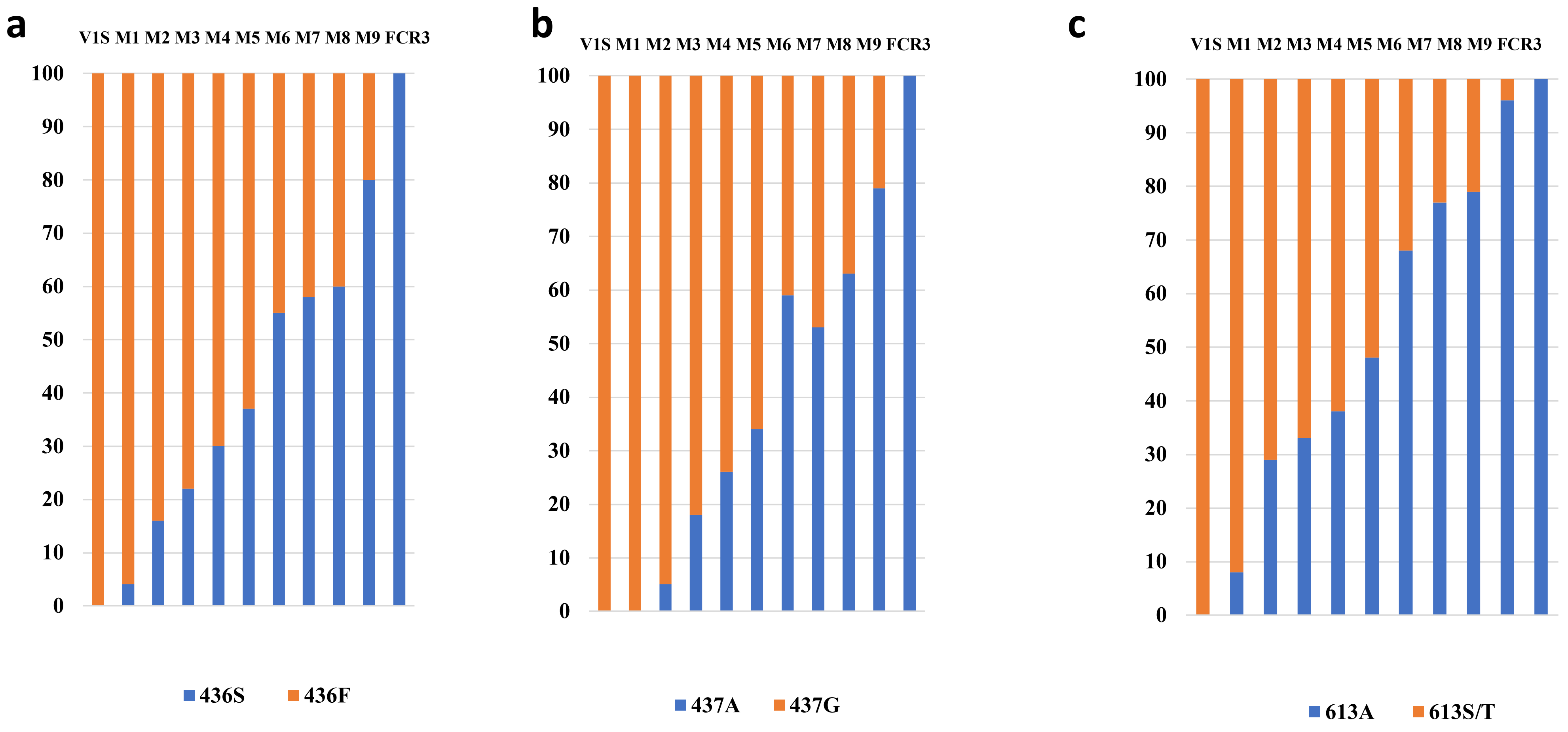

Supplementary Figure 6. Mean fraction and Prevalence of Pfdhps alleles at codons 436, 437 and 613

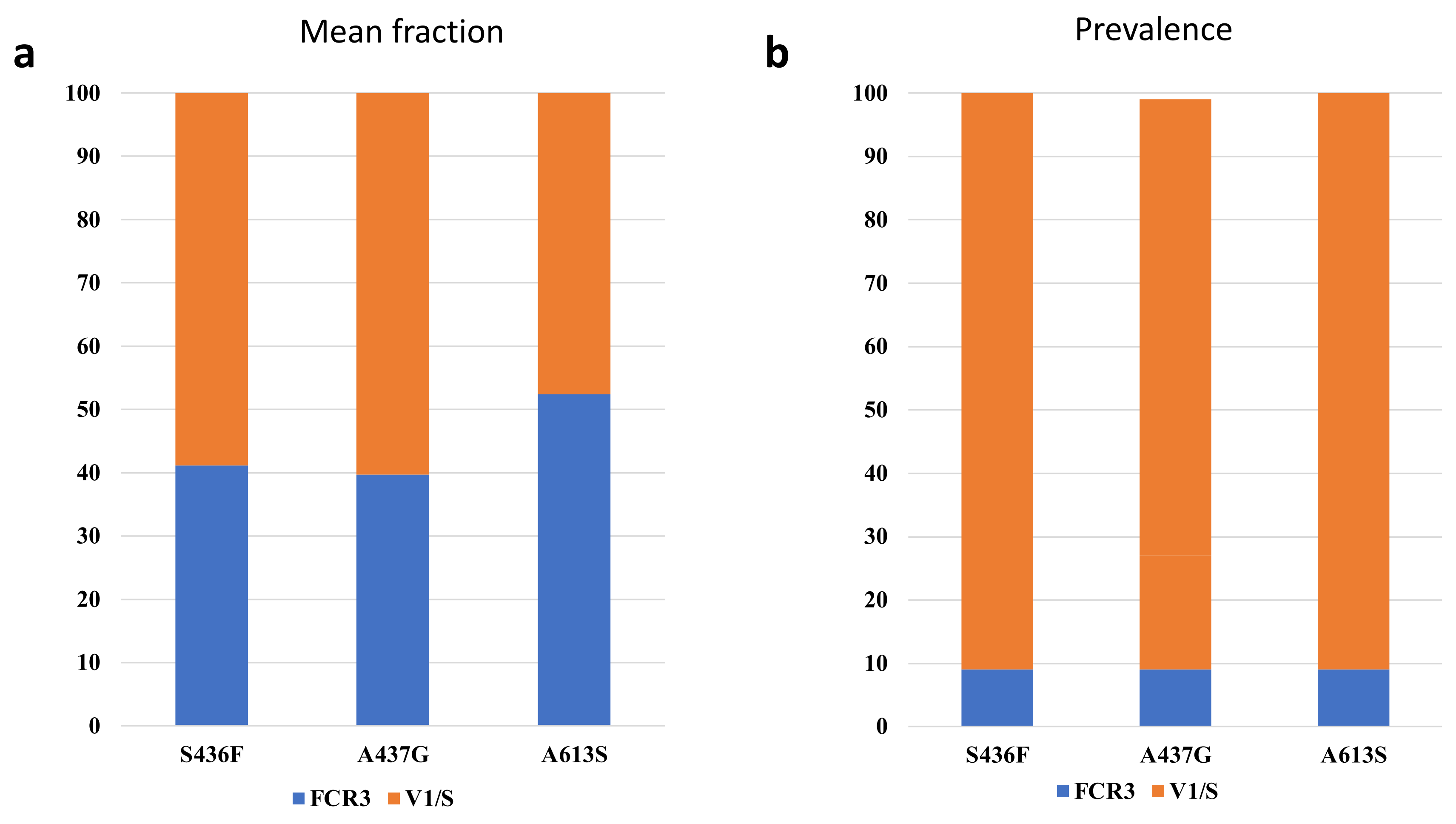

Supplementary Figure 7. Proportion of alleles of Pfdhfr at codons 51, 59 and 164

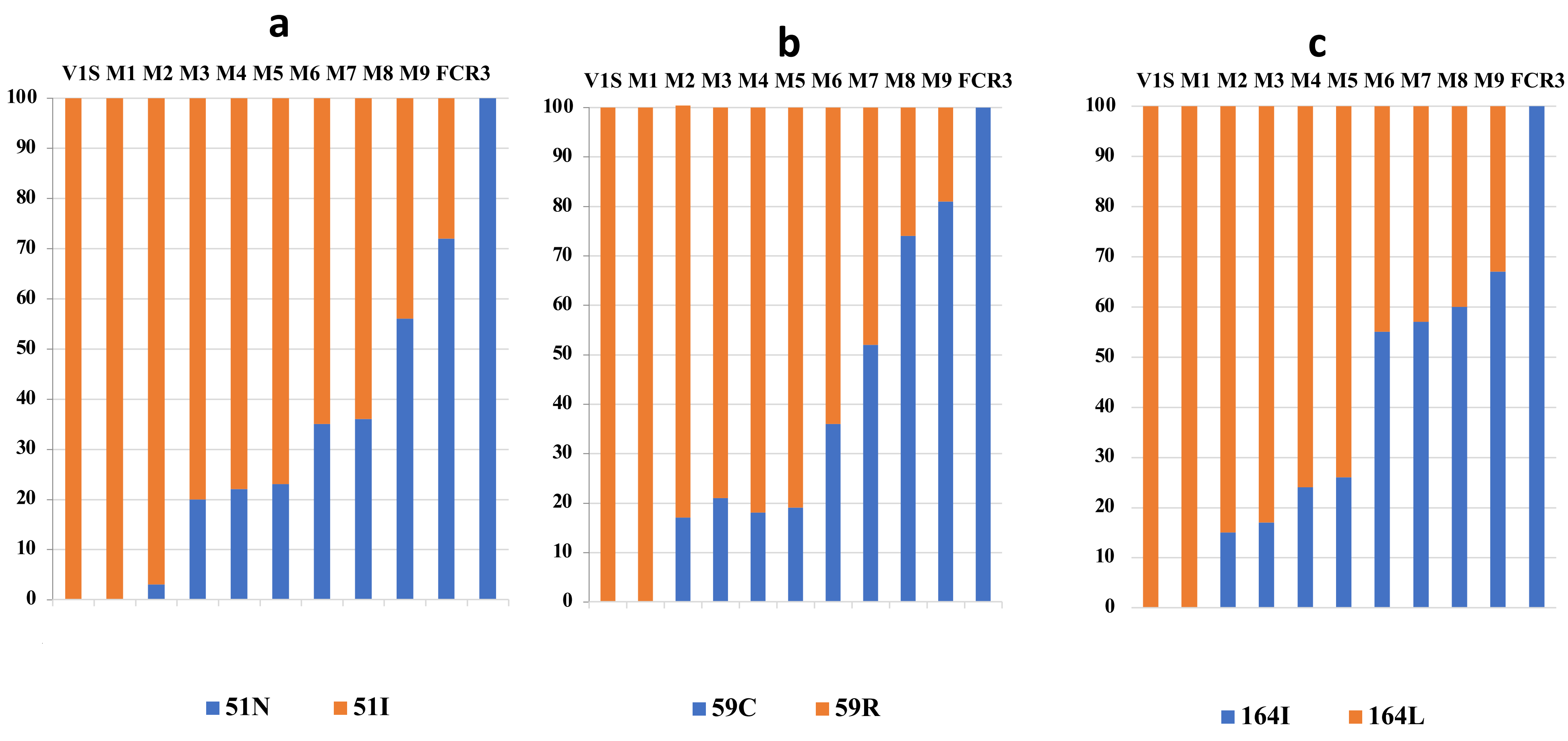

Supplementary Figure 8. Mean fraction and Prevalence of Pfdhfr alleles at codons 51, 59 and 164

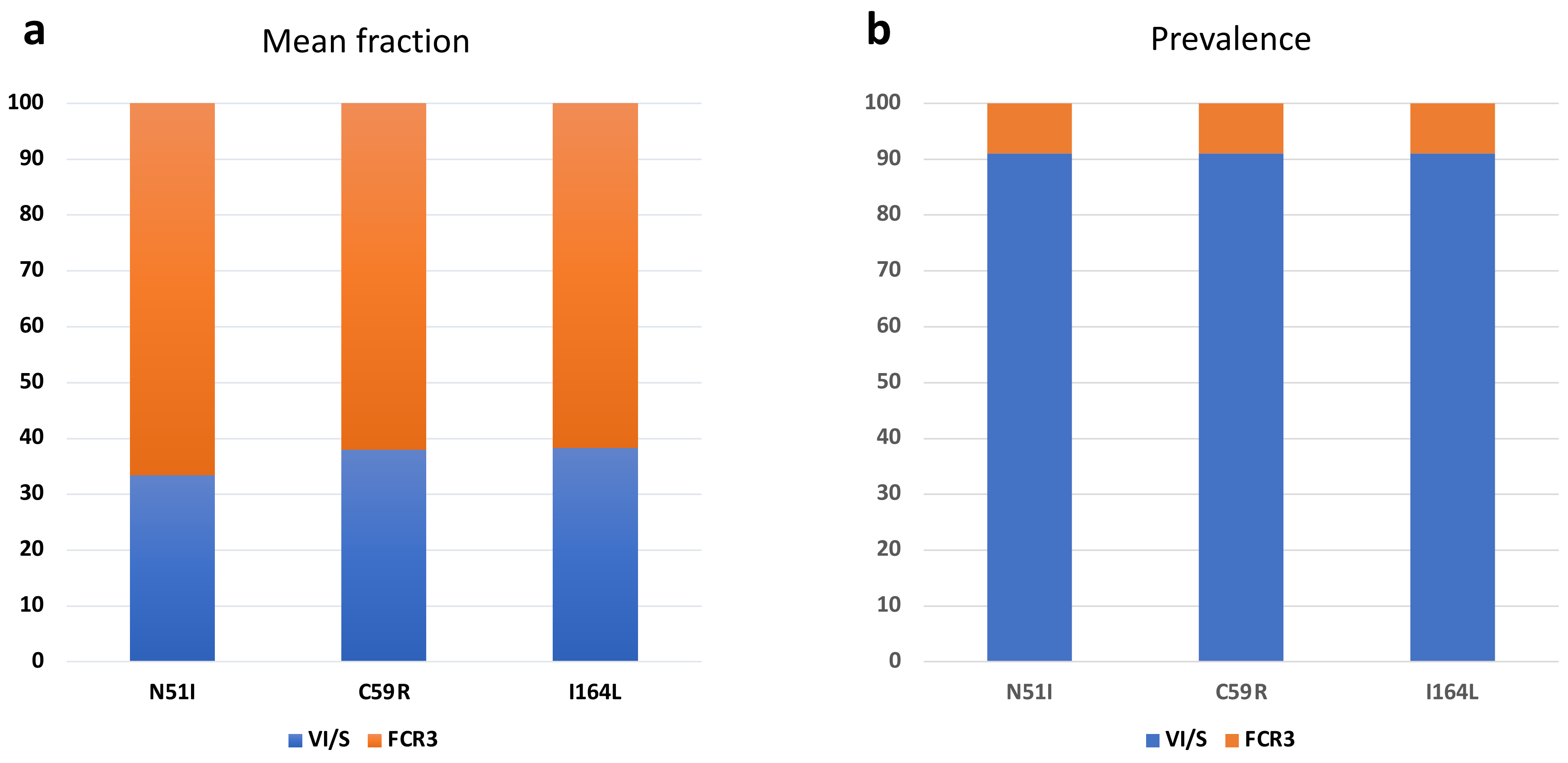

Supplementary Figure 9. Frequency of sensitive (S), resistant (R) and mix (M) alleles of Pfdhps

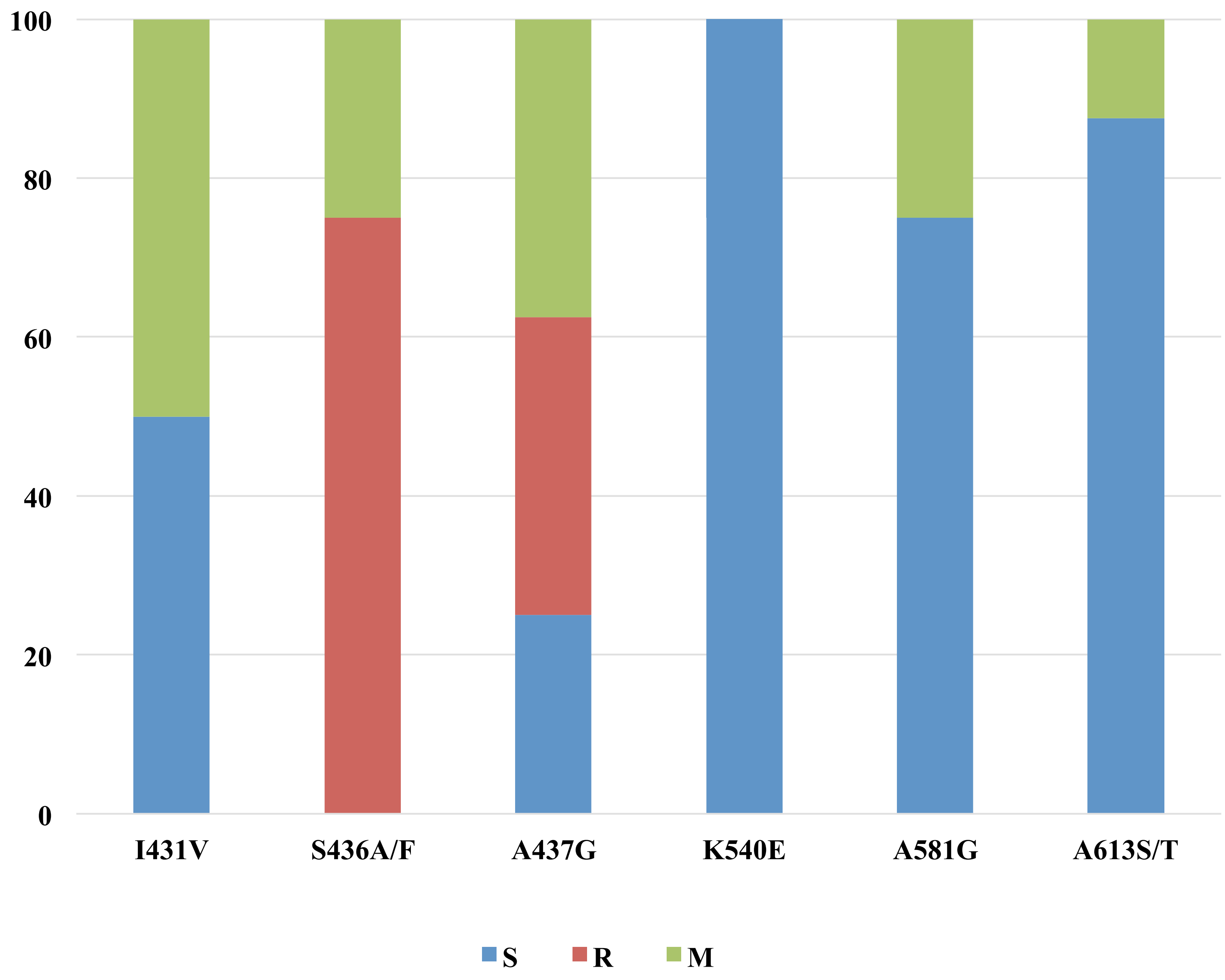

Supplementary Figure 10. Frequency of sensitive (S), resistant (R) and mix (M) alleles of Pfdhfr

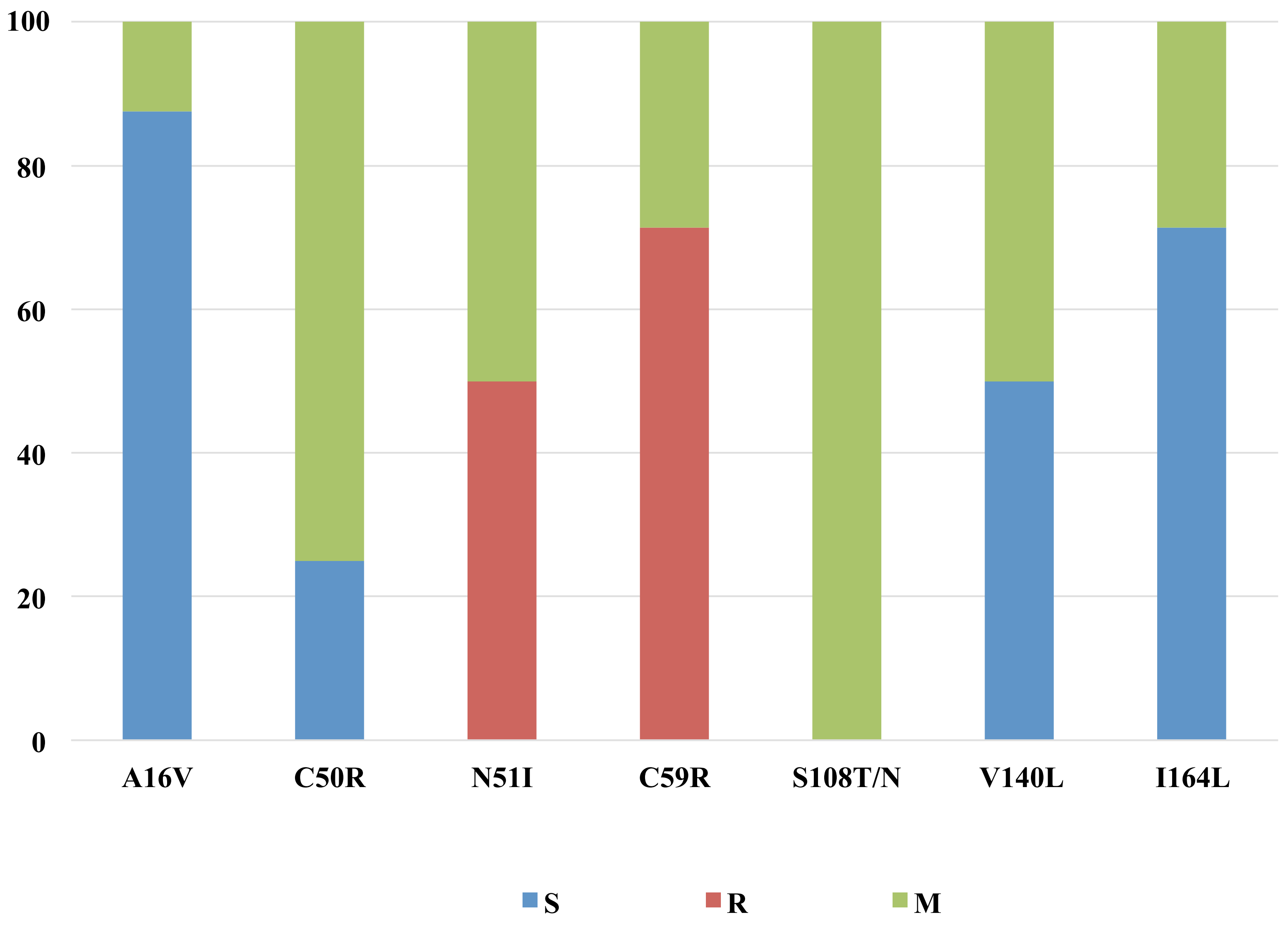

Supplement: Supplementary Figures [file mmc1.pdf]
